# Anti-Melanogenic Properties of Greek Plants. A Novel Depigmenting Agent from *Morus alba* Wood

**DOI:** 10.3390/molecules22040514

**Published:** 2017-03-23

**Authors:** Eliza Chaita, George Lambrinidis, Christina Cheimonidi, Adamantia Agalou, Dimitris Beis, Ioannis Trougakos, Emmanuel Mikros, Alexios-Leandros Skaltsounis, Nektarios Aligiannis

**Affiliations:** 1 Division of Pharmacognosy and Natural Product Chemistry, Department of Pharmacy, National & Kapodistrian University of Athens, Panepistimiopolis Zografou, GR-15771 Athens, Greece; elchaita@pharm.uoa.gr (E.C.); skaltsounis@pharm.uoa.gr (A.-L.S.); 2 Division of Pharmaceutical Chemistry, Department of Pharmacy, National & Kapodistrian University of Athens, Panepistimiopolis Zografou, GR-15771 Athens, Greece; lamprinidis@pharm.uoa.gr (G.L.); mikros@pharm.uoa.gr (E.M.); 3 Department of Cell Biology and Biophysics, Faculty of Biology, National & Kapodistrian University of Athens, GR-15784 Athens, Greece; chrischeim@biol.uoa.gr (C.C.); itrougakos@biol.uoa.gr (I.T.); 4 Developmental Biology, Biomedical Research Foundation Academy of Athens, Soranou Ephessiou 4, 11527 Athens, Greece; a.agalou@gmail.com (A.A.); dbeis@bioacademy.gr (D.B.)

**Keywords:** tyrosinase inhibition, Greek flora, *Morus alba*, dihydroxyresveratrol, 2,4,3′-trihydroxydihydrostilbene, melanogenesis, B16F10 melanoma cells, zebrafish, computational docking analysis

## Abstract

In therapeutic interventions associated with melanin hyperpigmentation, tyrosinase is regarded as a target enzyme as it catalyzes the rate-limiting steps in mammalian melanogenesis. Since many known agents have been proven to be toxic, there has been increasing impetus to identify alternative tyrosinase inhibitors, especially from natural sources. In this study, we investigated 900 extracts from Greek plants for potential tyrosinase inhibitive properties. Among the five most potent extracts, the methanol extract of *Morus alba* wood (MAM) demonstrated a significant reduction in intracellular tyrosinase and melanin content in B16F10 melanoma cells. Bioassay-guided isolation led to the acquisition of twelve compounds: oxyresveratrol (**1**), kuwanon C (**2**), mulberroside A (**3**), resorcinol (**4**), dihydrooxyresveratol (**5**), *trans*-dihydromorin (**6**), 2,4,3′-trihydroxydihydrostilbene (**7**), kuwanon H (**8**), 2,4-dihydroxybenzaldehyde (**9**), morusin (**10**), moracin M (**11**) and kuwanon G (**12**). Among these, 2,4,3′-trihydroxydihydrostilbene (**7**) is isolated for the first time from *Morus alba* and constitutes a novel potent tyrosinase inhibitor (IC_50_ 0.8 ± 0.15). We report here for the first time dihydrooxyresveratrol (**5**) as a potent natural tyrosinase inhibitor (IC_50_ 0.3 ± 0.05). Computational docking analysis indicated the binding modes of six tyrosinase inhibitors with the aminoacids of the active centre of tyrosinase. Finally, we found both MAM extract and compounds **1**, **6** and **7** to significantly suppress in vivo melanogenesis during zebrafish embryogenesis.

## 1. Introduction

The degree and distribution of melanin pigmentation are the main regulators of the mammalian skin and hair color [1]. Moreover, melanin plays a crucial role in oxidative homeostasis and protection of the skin from ionizing radiation [2,3]. However, abnormal accumulations of melanin pigments may result in various dermatological disorders, such as melasma, postinflammatory lesions and solar lentigines [4,5]. In therapeutic interventions associated with melanin hyperpigmentation, tyrosinase is regarded as a target enzyme, as it catalyzes the rate-limiting steps in mammalian melanogenesis. Tyrosinase catalyzes the hydroxylation of tyrosine to 3,4-dihydroxyphenylalanine (l-DOPA) (monophenolase activity) and the subsequent oxidation of this *o*-diphenol to the corresponding quinone, dopaquinone (diphenolase activity). From this point on, melanin is formed by a combination of enzymatically catalyzed and chemical reactions [2]. Over the past few years, tyrosinase has attracted an increasing interest of researchers and a number of potent inhibitors of synthetic, semi-synthetic and natural origin have been reported in an effort to develop preparations that suppress melanogenesis [6,7,8]. Since many known agents have been proven toxic (e.g., reports of hydroquinone for potential mutagenicity and epidemics of ochronosis [3]), there has been increasing impetus to find alternative tyrosinase inhibitors (especially from natural sources) for use in the cosmeceutical industry. Although natural occurring compounds have received wide acceptance, whole plant extracts have often demonstrated better or broader skin protective properties due to their complex composition [9]. Beyond these applications, tyrosinase inhibitors also constitute an emerging area in the food industry, as they are used to prevent enzymatic browning in fruits and vegetables. Moreover, the presence of the oxidation products of l-tyrosine has recently been linked to the demise of neurons in several neurodegenerative disorders such as Parkinson’s and Huntington’s diseases [10,11,12]. Thus, the identification of novel tyrosinase inhibitors is clearly important for both the development of therapeutic agents, as well as for new antioxidants and cosmeceuticals. 

Nevertheless, and despite the existence of a large number of tyrosinase inhibitors that were proven to be effective at in vitro assays, only a few were able to induce similar effects in clinical trials. In most cases, successful treatments need the combination of two or more agents acting on different mechanisms to achieve a synergistic effect [13]. Specifically, skin pigmentation is determined by various physiological processes where some stages are completely controlled genetically, while other stages are targets for manipulation of the cellular content [14]. Zebrafish is a powerful model to functionally test in vivo the activity of several chemical compounds [15] and to follow developmental phenotypes at a cellular resolution [16]. Embryonic melanocytes in zebrafish develop from neural crest-derived progenitors, migrate and differentiate by 48 h post fertilization (hpf). Differences in patterning, cell number and migration have been extensively used to screen for small molecules that inhibit melanogenesis [17].

In the past, biological screening procedures of extracts and/or pure compounds derived from plants of high biodiversity areas have been proven useful for the rapid identification of bioactive molecules [18,19]. The Greek flora presents great biodiversity, with approximately 6000 plant species growing in the region, of which ~1200 are endemic [20]. The country is located in the center of the Mediterranean basin, one of the 25 globally recognized hotspot regions, and is characterized by intense biodiversity. In an effort to discover novel anti-melanogenic natural agents, we focused for this work on the Greek flora, known for plants with antioxidant and/or other therapeutic properties [21,22]. We established a plant inventory of approximately 450 species and subspecies belonging to 66 plant families, which were screened for tyrosinase inhibitory properties. The wood of *Morus alba* demonstrated the strongest anti-melanogenic potential. Bioassay-guided isolation led to the identification of twelve compounds, among them a new potent tyrosinase inhibitor.

## 2. Results

### 2.1. Tyrosinase Inhibition Properties of Greek Extracts

We prepared 900 extracts from 450 Greek plants belonging to 66 different plant families, including the most prevalent families in the Greek flora (e.g., Asteraceae, Boraginaceae, Cistaceae, Cruciferae, Euphorbiaceae, Geraniaceae, Guttiferae, Lamiaceae, Leguminosae, Liliaceae, Pinaceae, Rosaceae, Scrophulariaceae, Umbelliferae). Screening for tyrosinase inhibition properties of all extracts was performed using an initial concentration of 300 μg/mL and this revealed 99 extracts with weak tyrosinase inhibitory properties (20%–40% inhibition), 41 extracts with moderate (40%–70% inhibition), and 15 extracts with promising anti-melanogenic potential (Supplementary Material Table S1). Next, we calculated IC_50_ values for the most potent agents (Table 1). 

The *Morus alba* extract was found to be the most potent tyrosinase inhibitor among all extracts investigated. Both methanol (MAM) and ethyl acetate (MAE) extracts exhibited a dramatic inhibition of the enzyme’s activity, with estimated IC_50_ values of 0.4 ± 0.02 and 1.3 ± 0.1 μg/mL respectively, denoting a highly promising source of phytochemicals for the discovery of potent anti-melanogenic agents. In particular, the methanol extract of *Morus alba* was found to be ~5–fold more potent than the reference compound kojic acid. In addition, *Glycyrrhiza glabra* also demonstrated strong anti-melanogenic potential, as both extracts exhibited particularly low IC_50_ values (2.1 ± 0.1 and 4.7 ± 0.3 μg/mL). Furthermore, *Veratrum album* and *Pistacia lentiscus* var. *chia* demonstrated significant tyrosinase inhibition, whereas all the other extracts exhibited anti-melanogenic properties to a lesser extent. 

### 2.2. Cytotoxicity and Determination of Melanin Content and Cellular Tyrosinase Activity

**Figure 1:**
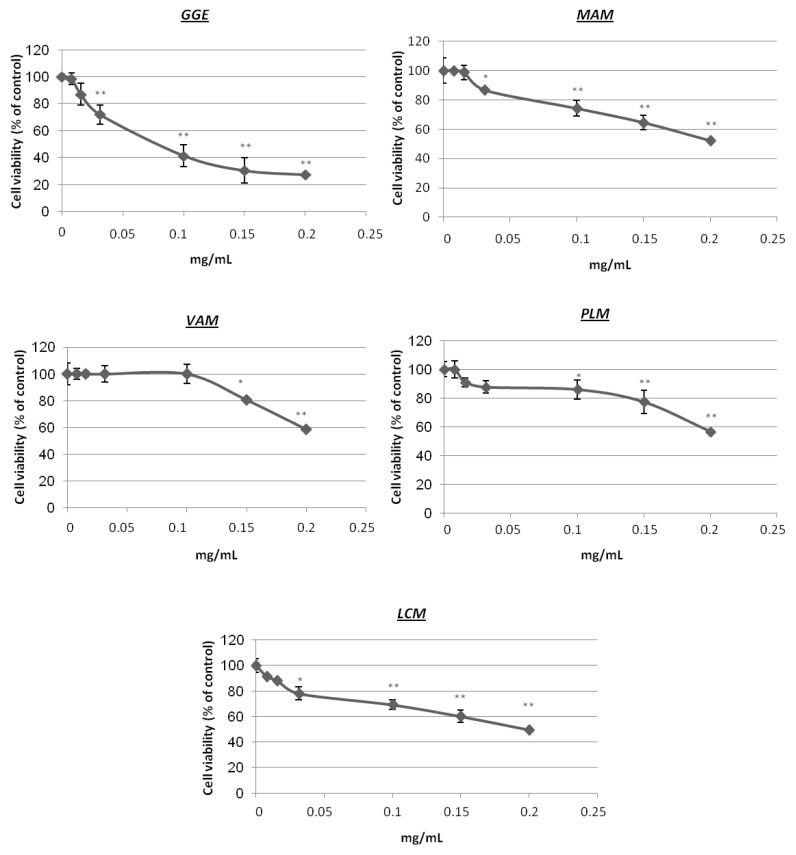
Relative (%) cell survival (MTT assay) of B16F10 cells after treatment with increasing concentrations of five selected extracts for 48 h; Bars, ±SD (*n* = 3). *, *p* < 0.05; **, *p* < 0.01 vs. controls set to 100%.

**Figure 2:**
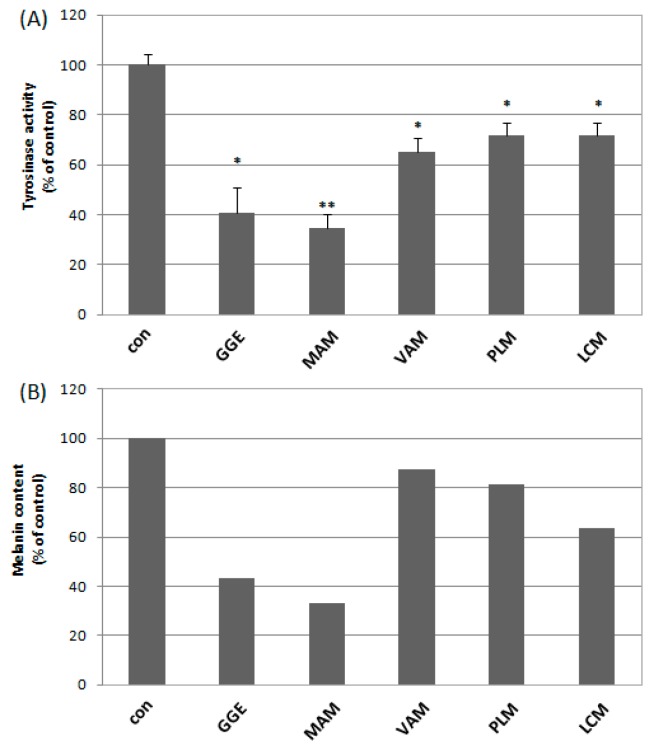
Effect of the five selected extracts on (**A**) intracellular tyrosinase activity inhibition (Bars, ±SD (*n* = 3) *, *p* < 0.05; **, *p* < 0.01 vs. controls set to 100%) and (**B**) on suppressing melanin cellular accumulation.

The ethyl acetate extract of *Glycyrrhiza glabra* (GGE) and methanol extracts of *Morus alba* (MAM), *Veratrum album* (VAM), *Pistacia lentiscus* var. *chia* (PLM) and *Lathyrus clymenum* (LCM) were further investigated in melanoma cell lines. We measured the potential cytotoxic properties of the extracts as well as their ability to alter tyrosinase activity in B16F10 melanoma cells. The extracts showed marginal toxicity to B16F10 cells with IC_50_ values reaching 0.2 mg/mL, with the exception of GGE that exhibited a slightly higher cytotoxicity, with an IC_50_ at 0.075 mg/mL. The relative survival of melanoma cells against all extracts is presented in Figure 1. In relation to the effect of the extracts on tyrosinase enzymatic activity (Figure 2), the MAM extract significantly reduced tyrosinase activity in B16F10 cells to ~35% of the control cells values. Similarly, GGE reduced tyrosinase activity of B16F10 cells at 40% (vs. controls); finally, VAM also reduced tyrosinase activity in B16F10 cells but to a lesser extent (~65% of the control values) as compared to the other extracts. LCM and PLM exhibited the same pattern of tyrosinase activity as VAM (~70% vs. control). Finally, in suppressing melanin cellular accumulation, MAM was the most effective plant extract (~30% of the values found in control cells), followed by GGE and LCM (Figure 2). On the basis of these cell-based assays, we selected the MAM extract for further investigation and isolation of the bioactive constituents.

### 2.3. Structure Elucidation of Isolated Compounds

**Figure 3:**
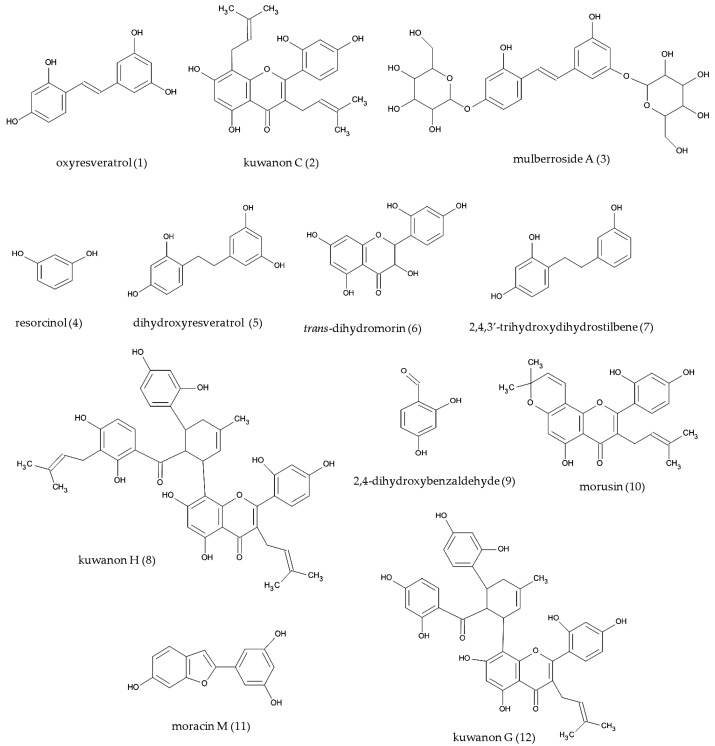
Compounds isolated from *Morus alba* wood.

Bioassay-guided isolation process led to the isolation of **12** compounds (Figure 3) from both extracts of *Morus alba*. Structural elucidation of all isolated compounds was performed by means of 1D and 2D NMR spectra. Comparison of the data with literature resulted in the identification of oxyresveratrol (**1**) [23], kuwanon C (**2**) [24], mulberroside A (**3**) [25], resorcinol (**4**) [26], dihydrooxyresveratol (**5**) [27], trans-dihydromorin (**6**) [28], 2,4,3′-trihydroxydihydrostilbene (**7**), kuwanon H (**8**) [29], 2,4-dihydroxybenzaldehyde (**9**) [30], morusin (**10**) [31], moracin M (**11**) [32] and kuwanon G (**12**) [29,33]. Compound **7** was isolated for the first time from *Morus alba* and has been reported only once in literature [34], without full spectroscopic data. The detailed spectroscopic data of the naturally occurring **5** are also presented for the first time.

### 2.4. Tyrosinase Inhibition of Isolated Compounds

Compounds derived from active fractions were screened for their inhibitory potential against diphenolase activity of enzyme tyrosinase initially in two concentrations, at 300 μM and 60 μM. Table 2 lists the inhibitory properties of the compounds, as well as the IC_50_ values determined for the most active inhibitors.

As is evident from these assays dihydrooxyresveratrol (**5**) exerted the highest tyrosinase inhibition effect, with an IC_50_ value of 0.3 ± 0.05 μM, approximately ~50-fold stronger than the reference control kojic acid (IC_50_ 16.1 ± 1.4 μM). As already mentioned, 2,4,3′-trihydroxydihydrostilbene (**7**) was isolated from *Morus alba* for the first time. The tyrosinase inhibitory properties of compound **7** were also investigated for the first time. It was found that the molecule represents a prominent tyrosinase inhibitor with IC_50_ value 0.8 ± 0.15 μM, demonstrating significantly stronger anti-melanogenic properties (~20-fold) as compared to kojic acid. *trans*-Dihydromorin (**6**) also demonstrated potent tyrosinase inhibition in the initial screening and the IC_50_ value of the flavanol was determined at 9.4 ± 1.3 μΜ. On the other hand, the flavones kuwanon C (**2**) and morusin (**10**) exhibited considerable tyrosinase inhibitory properties, but significantly lower than that of *trans*-dihydromorin. The inhibitory properties of kuwanon G (**12**) and H (**8**) against diphenolase activity of tyrosinase (IC_50_ 27.5 ± 1.2 μΜ and >100 μΜ, respectively) are reported for the first time. Dihydrooxyresveratrol (**5**) and 2,4,3′-trihydroxydihydrostilbene (**7**), the two dihydrostilbenes isolated from *Morus alba* proved to be stronger inhibitors than their stilbene analog, oxyresveratrol (IC_50_ 1.7 μM). This suggests that the reduction of the double bond in the stilbene structure may be responsible for the enhancement of the tyrosinase inhibitory properties, as the subsequent bibenzyl structure allows the molecule to approach and interact with the active center more effectively. In our previous study on the synthetic potential inhibitors of tyrosinase, we highlighted through molecular modeling studies the great importance of 2-OH, as well as of the 4-OH of dihydrostilbenes for the binding of the inhibitor at the entrance of the enzymes’s active centre [7]. Moreover, in the same study, the addition of 4′-OH on the B-ring was accompanied by a significant increase in the activity at *in vitro* tests; in support docking calculations showed a strong interaction with the receptor. A relevant question is whether the substitution of 4′-OH with the 3′-OH (on the B-ring in compound **7**), and the introduction of a second OH at position 5′ (in compound **5**), may relate to a more effective binding to the enzyme. For this reason, we examined this hypothesis by conducting molecular docking calculations.

### 2.5. Molecular Docking on Mushroom Tyrosinase

In order to comprehend the mechanism by which the isolated compounds inhibit the enzymatic activity of tyrosinase, we set out to explore the chemical groups of strong inhibitors, which interact with the active site of the enzyme. We performed docking calculations using the crystal structure of mushroom tyrosinase in complex with tropolone (PDB entry 2Y9X) [35]; furthermore, kuwanon C (2) (which demonstrated significantly lower inhibitory properties) was investigated.

**Figure 4:**
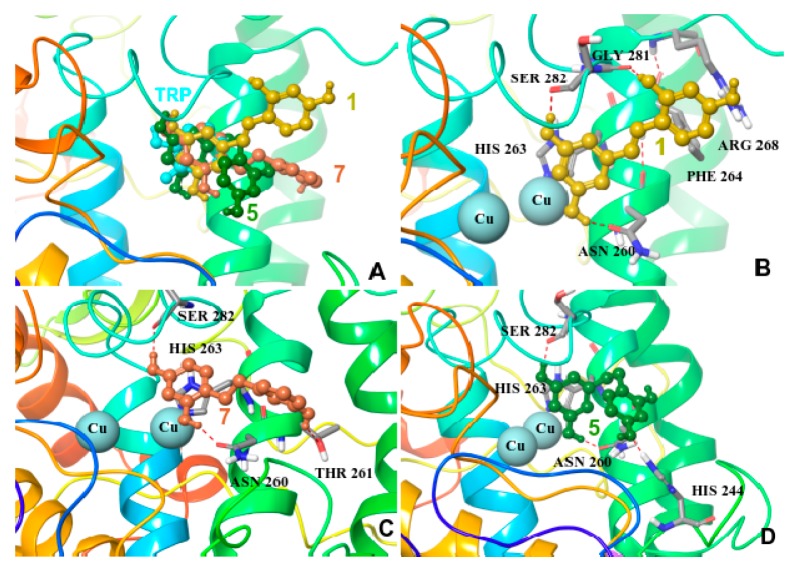
(**A**) Superposition of tropolone and low energy conformations of **1**, **5** and **7** inside the binding cavity of mushroom tyrosinase. Low energy structures of mushroom tyrosinase in complex with (**B**) oxyresveratrol (**1**); (**C**) 2,4,3′-trihydroxydihydrostilbene (**7**) and (**D**) dihydrooxyresveratrol (**5**).

Initially, the physicochemical properties for all compounds were evaluated. All potent inhibitors oxyresveratrol (**1**), dihydrooxyresveratrol (**5**), 2,4,3′-trihydroxydihydrostilbene (**7**), showed theoretical clogP values of 2.48, 2.47 and 2.82, respectively, as calculated by the Datawarrior software (http://www.openmolecules.org/datawarrior/). The rest of compounds showed cLogP < 0.9 or cLogP > 5 revealing the appropriate physicochemical properties needed for binding to tyrosinase and explaining the differences in the observed IC_50_ values. Induced Fit Docking to the most potent inhibitors revealed their binding modes which could explain the difference in binding affinity as measured by IC_50_ values. Compared with the tropolone inhibitor (as depicted from the crystal structure with mushroom tyrosinase) oxyresveratrol (**1**), dihydrooxyresveratrol (**5**), 2,4,3′-trihydroxy-dihydrostilbene (**7**) are bound using their 3,5 or/and 2,4 dihydroxyphenyl substitution near the catalytic site (Figure 4A) whereas the phenyl ring is fully superimposed with the tropolone ring. In all three compounds, hydrogen bonds are formed with ASN 260, which has been identified as crucial for inhibition in a previously conducted research by our group [7]. Additionally, **1** is stabilized by hydrogen bonds with Ser282 (backbone), His263, Gly281 (backbone) and also p-p stacking interaction with Phe264 and p-cation interaction with Arg268 (Figure 4B). Dihydrooxyresveratrol (**5**) is stabilized by hydrogen bond interaction with His 244, Ser 282 (backbone) and His 263 (Figure 4D). Finally, **7** is stabilized by hydrogen bond interaction with Thr261, Ser 282 (backbone) and His 263. (Figure 4C).

Moracin M (**11**), kuwanon C (**2**), and *trans*-dihydromorin (**6**) (*S,S* or *R,R*), are also bound to the same site of the protein. However, there are small differences which could explain the lower binding affinity. Moracin M (**11**), possesses a 3,5-dihydroxy substitution pattern, and approaches and binds near the catalytic center and the theoretical value of cLogP is 2.82, possibly because due to its rigidity the molecule cannot interact with Asn 260 which has been identified as crucial for inhibition (Supplementary Figure S1). On the other hand, kuwanon C (**2**) although it binds in a similar way and shows interaction with Asn 260, the prenyl groups are found near hydrophilic aminoacids (His, Asn, Arg) which lowers their binding affinity (Supplementary Figure S2). Additionally, the theoretical clogP of 2 is 6.17, which is unfavorable inside a hydrophilic binding pocket. A very interesting case ocurrs in the binding mode of *trans*-dihydromorin (**6**) (*S,S* or *R,R*). Although the ligand forms interactions that stabilize the molecule in the binding site (Supplementary Figures S2, S3), the clogP value is very low (0.9) compared to the most potent inhibitors **1**, **5**, and **7** and therefore it shows higher hydrophilicity. Thus, the unfavorable desolvation entropy term raises the free energy of molecule **6** binding to tyrosinase.

### 2.6. In Vivo Inhibition of Melanogenesis Assay

**Figure 5:**
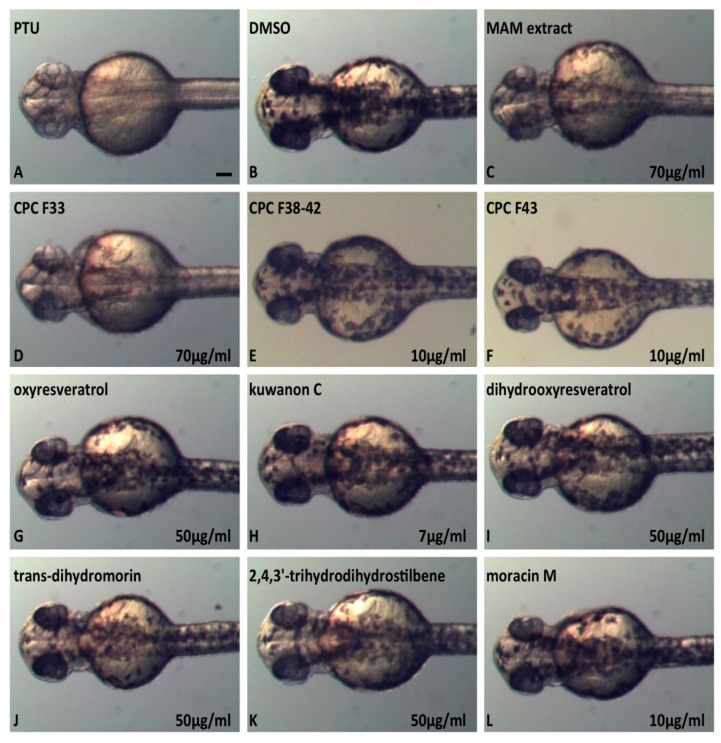
Melanogenesis inhibition on zebrafish embryos treated from 24 hpf to 48 hpf with *Morus alba* compounds. Bright field images of embryos treated with (**A**) PTU used as a positive control of melanogenesis inhibition; (**B**) DMSO used as negative control; (**C**) 70 μg/mL *Morus alba* methanol wood extract (MAM); (**D**) 70 μg/mL CPC F33 fraction that contains the compounds 2,4,3-trihydrodihydrostilbene (**7**), moracin M (**11**) and kuwanon G (**12**); (**E**) 10 μg/mL CPC F38-42 fraction that contains the compounds oxyresveratrol (**1**) and *trans*-dihydromorin (**6**); (**F**) 10 μg/mL CPC F43 fraction that contains the compounds oxyresveratrol (**1**) and dihydrooxyresveratrol (**5**); (**G**–**L**) purified compounds from CPC fractions: (**G**) 50 μg/mL oxyresveratrol (**1**); (**H**) 7 μg/mL kuwanon C (**2**); (**I**) 50 μg/mL dihydrooxyresveratrol (**5**); (**J**) 50 μg/mL *trans*-dihydromorin (**6**); (**K**) 50 μg/mL 2,4,3′-trihydrodihydrostilbene (**7**); (**L**) 10 μg/mL moracin M (**11**); Scale bar: 100μM.

In order to evaluate the inhibition of melanogenesis by the *Morus alba* extract and purified compounds in vivo, we used the zebrafish model that has been established as a new animal system for evaluating the depigmenting activity of melanogenic regulatory compounds [36]. Initially, we tested the *Morus alba* methanol extract and found that it significantly reduces the melanogenesis of embryos treated from 24 to 48 h post fertilization (hpf) (Figure 5C). Subsequently, we analyzed 20 FCPC fractions and identified fractions 33, 38–42 and 43 as the most potent (Figure 5D–F). We finally performed a screening assay on zebrafish embryos with the purified compounds and concentrations ranging from 1 to 100 μg/mL to determine their LC_50_ and optimal melanogenesis inhibition concentrations. Oxyresveratrol (**1**) (Figure 5G), *trans*-dihydromorin (**6**) (Figure 5J) and 2,4,3′-trihydroxydihydrostilbene (**7**) (Figure 5K) were the most potent ones, followed by moracin M (**11**) (Figure 5L) and dihydrooxyresveratrol (**5**) (Figure 5I) while kuwanon C (**2**) (Figure 5H) was the weakest inhibitor of melanogenesis. These data correlate well with the IC_50_ values of tyrosinase inhibition, with the exception of dihydrooxyresveratrol, that only partially inhibited the melanogenesis of zebrafish embryos. Since all of these molecules have been selected as potent inhibitors of tyrosinase in a cell free system, these types of discrepancies could be attributed to the pharmacokinetic properties of the compounds and its bioavailability for the zebrafish embryos. In addition, it is worthwhile noticing that FCPC fractions, and especially CPC33, are more potent in vivo from the single molecules, suggesting a synergistic effect of the compounds that are present in these fractions.

## 3. Materials and Methods

### 3.1. Chemicals and Instrumentation

Mushroom tyrosinase (EC Number 1.14.18.1) and all the reagents used in the bioassays were purchased from Sigma-Aldrich (St. Louis, MO, USA). Solvents were purchased by Merck KGaA (Darmstadt, Germany) and Carlo-Erba reagents S.A.S. (Peypin, France). Extraction of plant materials was carried out in an accelerated solvent extractor (ASE) System 300, Dionex (Sunnyvale, CA, USA). Centrifugal partition chromatography was performed using a Kromaton FCPC A instrument (Annonay, France) equipped with a 200 mL column, adjustable rotation of 200–2000 rpm, and a C-660 fraction collector (BUCHI Labortechnik AG, Flawil, Switzerland). Thin-layer chromatography (TLC) was carried out on silica gel F254-precoated plates from Merck Millipore (Darmstadt, Germany). Sephadex-LH20 separation was performed using methanol as solvent. Spectroscopic measurements were conducted on a TECAN Infinite M200 Pro microplate reader (Tecan Group, Männedorf, Switzerland) employing the Magellan^TM^ software version 7.0 (Tecan Group, Männedorf, Switzerland). NMR spectra were recorded on a Advance III 600 MHz instrument (Bruker BioSpin, Rheinstetten, Germany) operating at 600 MHz and 150 MHz for ^1^H and ^13^C, respectively, equipped with a 5 mm broadband inverse detection probe (BBI). Tetramethylsilane (TMS) was used as internal standard. Mass spectrometric data were generated on a hybrid LTQ Orbitrap mass spectrometer (Thermo Scientific, Darmstadt, Germany) using an ESI source in negative mode.

### 3.2. Extract Preparation

Greek plants were collected from diverse floristic regions of the country, covering large part of the endemic flora of Greece, and identified by Dr Eleftherios Kalpoutzakis. Voucher specimens were deposited at the Department of Pharmacognosy and Natural Products Chemistry, Faculty of Pharmacy, University of Athens. The collection includes 66 plant families growing on Greek soil. In total, 450 species and subspecies were air-dried and pulverized. Ethyl acetate and methanol extracts were prepared separately using Accelerated Solvent Extraction (ASE). Approximately 20 g of pulverized plant part were extracted in two different circles. Pressure was controlled at 1500 PSI and temperature at 60 °C for methanol and 40 °C for ethyl acetate extracts. Static time per cycle was 15 min. Finally, organic solvent was evaporated under reduced pressure.

### 3.3. Characterization data of Compounds 5 and 7

*Dihydrooxyresveratrol* (**5**). Compound **5** was obtained as a white amorphous powder and assigned the molecular formula of C_14_H_14_O_4_ as established by ESI-HRMS at *m*/*z* 245.0821 [M − H]^−^, ^1^H-NMR (CD_3_OD), δ 6.80 (1H, d, *J* = 8.1 Hz, H-6), 6.30 (1H, d, *J* = 2.2 Hz, H-3), 6.20 (1H, dd, *J* = 8.1/2.2 Hz, H-5), 6.18 (2H, d, *J* = 2.2 Hz, H-2′, H-6′), 6.10 (1H, t, *J* = 2.2 Hz, H-4′), 2.73 (2H, m, H-7), 2.67 (2H, m, H-8). ^13^C-NMR (CD_3_OD), δ 159.1 (C-3′), 159.1 (C-5′), 157.1 (C-2), 157.1 (C-4), 146.3 (C-1′), 131.4 (C-6), 120.8 (C-1), 107.9 (C-2′), 107.9 (C-6′), 107.1 (C-5), 103.3 (C-3), 100.9 (C-4′), 37.7 (C-8), 32.8 (C-7).

*2,4,3′-Trihydroxydihydrostilbene* (**7**): Compound **7** was obtained as a white amorphous powder and assigned the molecular formula of C_14_H_14_O_3_ as established by ESI-HRMS at *m*/*z* 229.0873 [M − H]^−^, ^1^H-NMR (CD_3_OD), δ 7.07 (1H, t, *J* = 7.8 Hz, H-5′), 6.79 (1H, d, *J* = 8.0 Hz, H-6), 6.68 (1H, dt, *J* = 7.8/1.9 Hz, H-6′), 6.65 (1H, t, *J* = 1.9 Hz, H-2′), 6.59 (1H, dt, *J* = 7.8/1.9 Hz, H-4′), 6.30 (1H, d, *J* = 2.3 Hz, H-3), 6.20 (1H, dd, *J* = 8.0/2.3 Hz, H-5), 2.75 (2H, s, H-7, H-8). ^13^C-NMR (CD_3_OD), δ 158.6 (C-3′), 157.5 (C-2), 157.5 (C-4), 145.9 (C-1′), 131.7 (C-6), 130.2 (C-5′), 121.2 (C-1), 121.2 (C-6′), 116.6 (C-2′), 113.8 (C-4′), 107.5 (C-5), 103.5 (C-3), 38.2 (C-8), 33.5 (C-7).

### 3.4. Tyrosinase Inhibition Assay

In our experiments, we investigated the ability of plant extracts and natural compounds to inhibit the oxidation of l-DOPA (l-3,4-dihydroxyphenylalanine) to dopaquinone and subsequently to dopachrome by the enzyme tyrosinase employing a protocol from Masuda et al. [37] with slight modifications. Test samples were dissolved in DMSO to stock solutions of 10 mg/mL and were diluted in the proper concentration in phosphate buffer 1/15 M (NaH_2_PO_4_/Na_2_HPO_4_), pH 6.8; final concentrations of DMSO in the well did not exceeded 3%. In 96-well plates, 80 μL of phosphate buffer, 40 μL of sample in the same buffer and 40 μL mushroom tyrosinase (92 Units/mL), in the same buffer, were mixed. The contents of each well were incubated for 10 min at 25 °C, before the addition of 40 μL of 2.5 mM L-DOPA in phosphate buffer. After incubation at 25 °C for 5 min, the absorbance at 475 nm of each well was measured using a microplate reader. Blanks for every sample w/o tyrosinase were also performed, while kojic acid was used as positive control. The percentage inhibition of the tyrosinase activity was calculated by the following equation: [(A − B) − (C − D)]/(A − B) × 100, where A: Control (w/o sample), B: Blank (w/o sample, w/o tyrosinase), C: Sample, D: Blank sample (w/o tyrosinase).

### 3.5. Cell Lines and Cell Culture Conditions

Mouse skin melanoma (B16F10) cells were obtained from the American Tissue Culture Collection (Manassas, VA, USA) and were maintained in Dulbecco's modified Eagle’s medium (Gibco, Thermo Fischer Scientific, Waltham, MA, USA), supplemented with 10% (*v*/*v*) fetal bovine serum (Gibco, Thermo Fischer Scientific) and 2 mM glutamine (Gibco, Thermo Fischer Scientific) in a humidified incubator at 5% CO_2_ and 37 °C. 

#### 3.5.1. MTT Cytotoxicity Assay

MTT cytotoxicity assay was performed as described previously [38]. Briefly, B16F10 cells were cultured at 96-wellmicroplates (5000 cells/well). After 24 h the cells were treated with different concentrations of the extracts (in control cells an appropriate amount of DMSO was added). The MTT solution (MTT, Sigma-Aldrich) (1 mg/mL in serum free, phenol red free medium) was added 48 h after the addition of test extracts. After 4 h, the MTT solution was discarded and isopropanol was added to dissolve the formazan crystals. Absorbance was recorded at 570 nm using a microplate reader (EZ Read 400 ELISA microplate reader, Biochrom, Cambridge, UK). Survival of non-treated cells was set to 100%.

#### 3.5.2. Melanin Content Assay

Melanin content was assayed as described previously [39]. Briefly, B16F10 cells were cultured at 5 × 10^5^ cells/plate and were left to adhere. They were then treated with the extract samples (in control cells an appropriate amount of DMSO was added). After 48 h, cells were washed with PBS and were harvested with the use of trypsin-EDTA. Following centrifugation, the pellets were solubilized in 200 μL of 1 N NaOH at 95 °C for 1 h. The absorbance was measured at 405 nm using a microplate reader (EZ Read 400 ELISA microplate reader, Biochrom, Cambridge, UK). Melanin content was calculated using control cells as 100%. 

#### 3.5.3. Cellular Tyrosinase Activity Assay

Tyrosinase activity in B16F10 cells was assayed as described previously [40] with minor modifications. Briefly, cells were plated at a density of 25 × 10^3^ cells/well in 96 well plates and after 24 h they were treated with extract samples (in control cells an appropriate amount of DMSO was added). Following a period of 48 h exposure to extracts cells were washed with cold PBS and lysed with phosphate buffer (pH 6.8) containing 1% Triton-X/PBS (90 μL/well). They were then left to −80 °C for 30 min. After thawing and mixing, 100 μL of 0.1% L-DOPA was added to each well following incubation at 37 °C for 1 h. Absorbance was measured at 492 nm using the TECAN Infinite M200 Pro microplate reader employing the Magellan^TM^ software.

### 3.6. FCPC-Based Bioguided Isolation from Morus alba Extracts

Most promising extracts MAM and MAE (Table 1) were fractionated by Fast Centrifugal Partition Chromatography (FCPC) using a step-gradient elution-extrusion method. Two different series of five biphasic systems were employed consisting of heptane, ethyl acetate, butanol, ethanol and 10% acetic acid in water in ratio of 10/5/0/5/10, 5/10/0/5/10, 1/14/0/5/10, 1/12/2/5/10, 1/9/5/5/10 for the methanol (MAM) and 13/2/0/7/8, 10/5/0/7/8, 7/8/0/7/8, 4/11/0/7/8, 1/14/0/7/8 for the ethyl acetate (MAE) extract. Organic phase was used as mobile phase in increased polarity order. Flow was kept constant at 5 mL/min. Rotation of the column increased gradually during the analysis starting from 850 rpm. First, 40 mL of the first mobile phase were discarded and 160 mL of organic phase were collected at every step of the mobile phase series systems. Collection volume was 10 mL and 96 fractions were generated from this procedure. Resulting fractions were evaporated to dryness using a Genevac evaporator (Genevac Ltd, Suffolk, UK), transferred into 96-deep well plates, and submitted to tyrosinase inhibition assay and thin layer chromatography for investigation of their anti-pigmenting properties and phytochemical profile, respectively. Bioassay-guided isolation process led to the isolation of 12 compounds. Four compounds were isolated directly during FCPC fractionation and eight more were purified from active fractions. Compounds **1** and **2** were isolated directly during FCPC fractionation from both extracts, in fractions 70–71 and 45 respectively from MAE, as well as 43–44 and 17–19 respectively from MAM. Compound **3** was also obtained directly from FCPC fractions 63–64 from MAM, whereas compound **10** was isolated in fractions 17–19 during MAE fractionation. Fractions 38–42 of MAM extract that demonstrated strong anti-tyrosinase activity were submitted to Sephadex LH-20 separation to give compounds **4**, **5** and **6**, whereas **7** was purified utilizing preparative TLC from active fraction 62 of MAE. Preparative TLC led to the acquisition of compound **9** from fraction 34–35 of MAE, as well as **11**, **12** and **8** from active fractions 65 and 55–58 of MAE, respectively. In total, the conduction of two parallel FCPCs, one for the methanol and one for the ethyl acetate extract, led to the direct isolation of four compounds while aided significantly the purification process. Identification of isolated compounds was assessed by nuclear magnetic resonance (NMR) spectroscopy and mass spectrometry.

### 3.7. Molecular Docking on Mushroom Tyrosinase

The mushroom tyrosinase structure was retrieved from the Protein Data Bank (PDB entry 2Y9X). The protein was prepared for the docking calculations using the Protein Preparation Workflow (Schrödinger Suite 2011 Protein Preparation Wizard) implemented in the Schrödinger suite and accessible from within the Maestro program (Maestro, version 9.2, Schrödinger, LLC, New York, NY, USA, 2011). Ligand preparation for docking was performed with the LigPrep application (LigPrep, version 2.5, Schrödinger LLC). Molecular docking was performed using the Induced Fit Docking (IFD) protocol (Schrödinger Suite 2011 Induced Fit Docking protocol). CLogP values were calculated using Datawarrior software (http://www.openmolecules.org/datawarrior/).

### 3.8. Zebrafish Maintenance, Breeding and Exposure

Zebrafish embryos were raised under standard laboratory conditions at 28 °C in the zebrafish breeding establishment of the Biomedical Research Foundation of the Academy of Athens (EL25BIO001) as described previously [41]. Zebrafish are maintained in accordance with the European Directive 2010/63 for the protection of animals used for scientific purposes and the Recommended Guidelines for Zebrafish Husbandry Conditions. (https://www.eufishbiomed.kit.edu/59.php). The experimental protocols described in this study were carried out with zebrafish larvae up to 72 h post fertilization (hpf) and therefore are not subject to the regulations of European animal protection guidelines. The genetic background used was the wild-type AB strain. For the melanogenesis inhibition experiments, synchronized 24 hpf embryos were exposed to different concentrations of *Morus alba* extracts and purified compounds dissolved in DMSO in embryo water. Treatments were done in three independent experiments with 20 embryos per experimental sample. Images were acquired at 48 hpf with a DFK22BUC03 camera from The Imaging Source (Bremen, Germany), under a SMZ2800 stereoscope (Nikon, Tokyo, Japan).

## 4. Conclusions

To the best of our knowledge, this is the first large scale screening of Greek flora for anti-hyperpigmenting properties. Fifty-six extracts demonstrated considerable tyrosinase inhibitory activity, introducing a significant field for the discovery of promising compounds with anti-melanogenic effect. The methanol extract of *Morus alba* wood demonstrated the highest tyrosinase inhibition among the 900 extracts tested, as well as a significant reduction in intracellular tyrosinase and melanin content in B16F10 melanoma cells. Bioassay-guided isolation led to the acquisition of twelve compounds, including five with significant tyrosinase inhibition (1.7-fold to 53-fold compared to the positive control kojic acid). 2,4,3′-Trihydroxydihydrostilbene (**7**), that was isolated for the first time from *M. alba*, is a novel tyrosinase inhibitor with 20-fold more potent inhibitory properties than kojic acid, rendering its chemical scaffold a strong candidate for the development of new potent anti-hyperpigmenting agents. Computational docking analysis indicated the binding modes of **7** (and five more tyrosinase inhibitors) with the aminoacids of the active centre of tyrosinase. Furthermore, both the MAM extract and compound **7** were found to be non-cytotoxic in high concentrations in cells, and significantly suppressed in vivo melanogenesis in zebrafish embryogenesis. These findings confirm the use of *Morus alba* as a valid source of anti-melanogenic agents. Overall, our results suggest that 2,4,3′-trihydroxydihydrostilbene (**7**), can be considered as a promising candidate for the treatment of dermatological disorders associated with melanin pigments. Other promising extracts obtained from Greek flora species will be further investigated for the discovery of novel compounds with anti-hyperpigmenting properties.

## Figures and Tables

**Table 1:** Tyrosinase inhibition of the 15 most prominent extracts from Greek plants.

Plant Species	Plant Family	Plant Part	Extraction Solvent	Tyrosinase Inhibition		
				300 μg/mL	75 μg/mL	IC_50_ (μg/mL) *
*Morus alba*	Moraceae	wood	MeOH	97	98	0.4 ± 0.02
*Morus alba*	Moraceae	wood	EtOAc	83	96	1.3 ± 0.1
*Glycyrrhiza glabra*	Leguminosae	roots	EtOAc	82	89	2.1 ± 0.1
*Glycyrrhiza glabra*	Leguminosae	roots	MeOH	92	88	4.7 ± 0.3
*Veratrum album*	Liliaceae	whole plant	MeOH	78	60	23.5 ± 3.8
*Pistacia lentiscus* var. *chia*	Anacardiaceae	branches & leaves	MeOH	76	58	62.0 ± 1.2
*Cistus salvifolius*	Cistaceae	aerial parts	MeOH	79	45	85.6 ± 10.8
*Umbilicus horizontalis*	Crassulaceae	whole plant	MeOH	81	51	86.6 ± 11.2
*Cistus salvifolius*	Cistaceae	aerial parts	EtOAc	71	41	95.3 ± 11.3
*Lathyrus clymenum*	Leguminosae	perisperm	MeOH	75	40	95.5 ± 2.1
*Sedum sediforme*	Crassulaceae	aerial parts	MeOH	70	40	116.6 ± 10.2
*Paeonia mascula* ssp *hellenica*	Paeoniaceae	aerial parts	MeOH	70	42	135.2 ± 12.2
*Armeria canescens*	Plubaginaceae	whole plant	MeOH	76	43	138.1 ± 9.0
*Cercis siliquastrum*	Leguminosae	aerial parts	MeOH	70	25	>150
*Pistacia terebinthus*	Anacardiaceae	leaves & stems	MeOH	70	35	>150
*kojic acid*						1.9 ± 0.1

* IC_50_ values are expressed as mean values ± STD of three different experiments.

**Table 2:** Tyrosinase inhibition in 300 μM and 60 μM, and IC_50_ values of compounds isolated from *Morus alba* wood.

Compound	Tyrosinase Inhibition		
	% ± STD (300 μM)	% ± STD (60 μM)	IC_50_ ± STD (μΜ) *
Oxyresveratrol (**1**)	97.8 ± 0.1	93.1 ± 0.1	1.7 ± 0.04
Kuwanon C (**2**)	75.6 ± 1.2	46.5 ± 1.3	76.2 ± 1.1
Mulberroside A (**3**)	58.3 ± 1.4	43.1 ± 0.7	>100
Resorcinol (**4**)	60.4 ± 1.0	38.0 ± 1.1	162.6 ± 18.2
Dihydrooxyresveratrol (**5**)	98.2 ± 0.5	97.6 ± 0.2	0.3 ± 0.05
*trans*-Dihydromorin (**6**)	94.3 ± 0.1	73.1 ± 0.6	9.4 ± 1.3
2,4,3′-Trihydroxydihydrostilbene (**7**)	97.8 ± 0.1	96.3 ± 0.5	0.8 ± 0.15
Kuwanon H (**8**)	49.0 ± 2.6	46.0 ± 2.9	>100
2,4-Dihydroxybenzaldehyde (**9**)	4.9 ± 0.3	na	na
Morusin (**10**)	49.9 ± 2.2	31.0 ± 3.2	>100
Moracin M (**11**)	87.2 ± 0.5	61.3 ± 1.2	8.0 ± 0.6
Kuwanon G (**12**)	77.8 ± 2.8	59.1 ± 1.4	27.5 ± 1.2
Kojic acid	96.0 ± 0.2	94.2 ± 0.2	16.1 ± 1.4

* IC_50_ values are expressed as means ± STD, for three independent experiments, screening at 300 μΜ and 60 μΜ are expressed as means ± STD, for two independent experiments; IC_50_ values were estimated for compounds that demonstrated >50% inhibition at 300 μM and exhibited a dose-dependent enzyme inhibition.

## Supplementary Materials

Supplementary Materials are available online.
