# Supplementary material for: Anti-Melanogenic Properties of Greek Plants. A Novel Depigmenting Agent from Morus alba Wood
Source: Molecules. 2017 Mar 23;22(4):514. doi: 10.3390/molecules22040514 (PMC6154579; doi:10.3390/molecules22040514)
Supplement: Supplementary file 1 [file molecules-22-00514-s001.pdf]

Supplementary Data

Table S1. Greek extracts that exhibited moderate and strong tyrosinase inhibition at initial concentration screens (300 μg/ml)

Plant species	Plant family	Plant part	Extraction solvent	% Tyrosinase inhibition
Abies cephalonica	Pinaceae	bark of stem and branches	MeOH	59
Acantholimon androsaceum	Plubaginaceae	aerial parts	MeOH	55
Acanthus spinosus	Acanthaceae	aerial parts	EtOAc	48
Anthyllis montana	Leguminosae	aerial parts	EtOAc	48
Anthyllis montana	Leguminosae	aerial parts	MeOH	46
Arbutus unedo	Ericaceae	stems and leaves	MeOH	50
Arbutus unedo	Ericaceae	stems and leaves	EtOAc	44
Armeria canescens	Plubaginaceae	aerial parts and roots	MeOH	76
Calicotome villosa	Leguminosae	aerial parts	MeOH	44
Ceratonia siliqua	Leguminosae	stems and leaves	MeOH	57
Ceratonia siliqua	Leguminosae	stems and leaves	EtOAc	44
Cercis siliquastrum	Leguminosae	aerial parts	MeOH	70
Chamaecytisus austriacus	Leguminosae	aerial parts	MeOH	46
Chamaecytisus triflorus	Leguminosae	aerial parts	MeOH	47
Chamaecytisus triflorus	Leguminosae	aerial parts	EtOAc	40
Cistus creticus ssp. creticus	Cistaceae	aerial parts	MeOH	63
Cistus creticus ssp. eriocephalus	Cistaceae	aerial parts	MeOH	58
Cistus monspeliensis	Cistaceae	aerial parts	MeOH	41
Cistus parviflorus	Cistaceae	aerial parts	MeOH	68
Cistus salvifolius	Cistaceae	aerial parts	MeOH	79
Cistus salvifolius	Cistaceae	aerial parts	EtOAc	71
Cotinus coggygria (Rhus cotinus)	Anacardiaceae	stems and leaves	MeOH	43
Cupressus sempervirens (forma horizontalis)	Cupressaceae	branches and leaves	EtOAc	47
Cytisus villosus	Leguminosae	aerial parts	MeOH	49
Dorycnium hirsutum	Leguminosae	aerial parts	MeOH	56
Dorycnium hirsutum	Leguminosae	aerial parts	EtOAc	44
Eryngium amorginum	Umbelliferae	aerial parts	EtOAc	56
Genista depressa	Leguminosae	aerial parts and roots	EtOAc	53
Geranium macrorrhizum	Geraniaceae	aerial parts and roots	MeOH	55
Glycyrrhiza glabra	Leguminosae	roots	MeOH	92
Glycyrrhiza glabra	Leguminosae	roots	EtOAc	82
Heracleum sphondylium ssp. pyrenaicum	Umbelliferae	aerial parts	EtOAc	43
Hippocrepis comosa	Leguminosae	aerial parts	MeOH	55
Hippocrepis comosa	Leguminosae	aerial parts	EtOAc	54

Lathyrus clymenum	Leguminosae	perisperm	MeOH	92
Lathyrus clymenum	Leguminosae	perisperm	EtOAc	59
Mentha pulegium	Lamiaceae	aerial parts	EtOAc	43
Morus alba	Moraceae	wood	MeOH	97
Morus alba	Moraceae	wood	EtOAc	83
Myrtus communis	Myrtaceae	aerial parts	MeOH	45
Onobrychis alba ssp. laconica	Leguminosae	aerial parts and roots	EtOAc	43
Onobrychis peloponnesiaca	Leguminosae	roots	EtOAc	42
Paenia clusii ssp. clusii	Paeoniaceae	aerial parts	MeOH	49
Paenia mascula ssp. hellenica	Paeoniaceae	aerial parts	MeOH	68
Pinus nigra ssp. pallasiana	Pinaceae	bark	MeOH	40
Pistacia lentiscus	Anacardiaceae	branches and leaves	MeOH	76
Pistacia lentiscus	Anacardiaceae	wood	MeOH	57
Pistacia terebinthus	Anacardiaceae	leaves and stems	MeOH	70
Pistacia terebinthus	Anacardiaceae	branches and leaves	MeOH	67
Polygonum idaeum	Leguminosae	aerial parts and roots	MeOH	59
Saxifraga sp.	Saxifragaceae	aerial parts and roots	MeOH	45
Sedum sediforme	Crassulaceae	aerial parts	MeOH	70
Sinapis alba	Cruciferae	aerial parts	EtOAc	41
Umbilicus horizontalis	Crassulaceae	aerial parts and roots	MeOH	81
Veratrum album	Liliaceae	aerial parts and roots	MeOH	78
Veratrum album	Liliaceae	aerial parts and roots	EtOAc	59

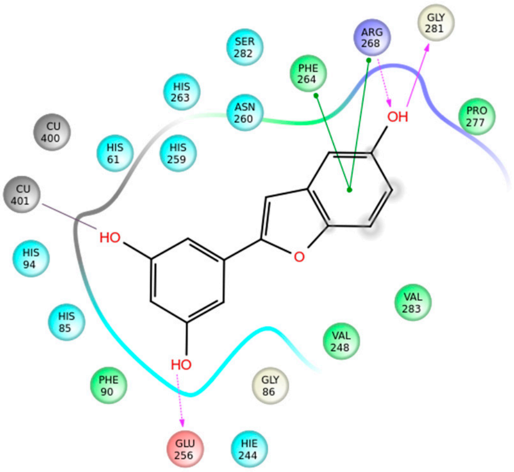

Supplementary Figure S1: Ligand Interaction Diagram of Low Energy Structure of Mushroom Tyrosinase in complex with **11**

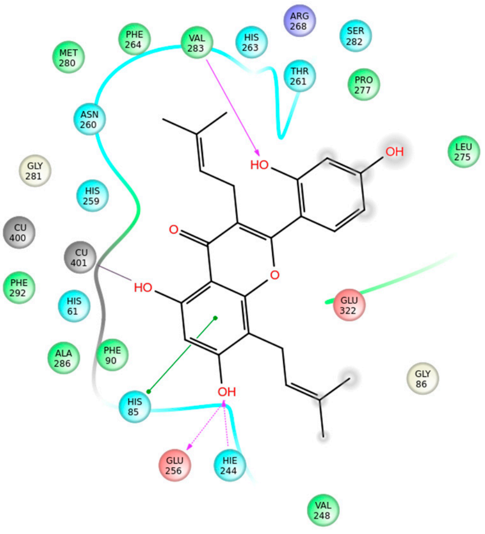

Figure S2: Ligand Interaction Diagram of Low Energy Structure of Mushroom Tyrosinase in complex with **2**

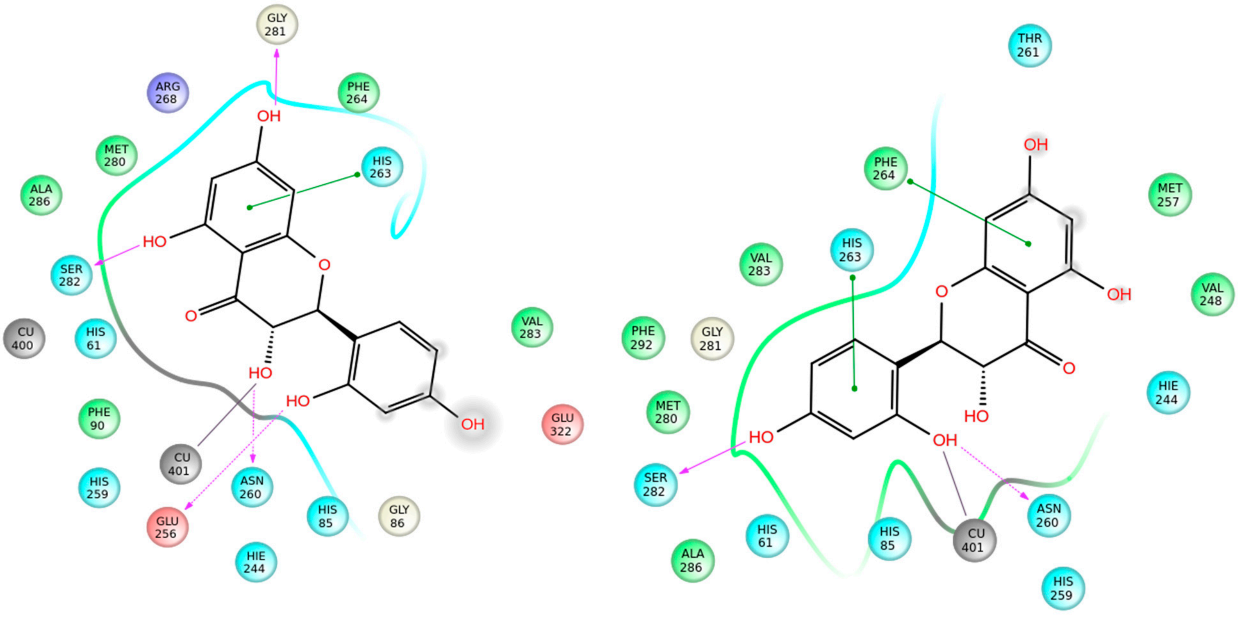

Figure S3: Ligand Interaction Diagram of Low Energy Structure of Mushroom Tyrosinase in complex with **6,** RR enantiomer (left) and SS enantiomer (right).
